# Results of cervical epidural steroid injections based on the physician referral source

**DOI:** 10.1016/j.inpm.2021.100001

**Published:** 2021-12-06

**Authors:** Josh Levin, Nolan Gall, John Chan, Lisa Huynh, Jayme Koltsov, D.J. Kennedy, Matt Smuck

**Affiliations:** a Department of Orthopaedic Surgery, Stanford University, 450 Broadway St., Pavilion C, 4th Floor, MC 6342, Redwood City, CA, 94063, United States; b Department of Neurosurgery, Stanford University, United States; c Department of Physical Medicine and Rehabilitation, Vanderbilt University, 2201 Children’s Way, Suite 1318, Nashville, TN, 37212, United States

**Keywords:** Cervical, Transforaminal, Interlaminar, Epidural, Referral source

## Abstract

**Background:**

Many patients who receive cervical epidural steroid injections (ESIs) are referred for the injection from a physician who does not perform the procedure.

**Purpose:**

To compare success rates of fluoroscopically-guided cervical ESIs in patients who had a clinical evaluation and recommendation for the injection by a fellowship-trained spine specialist who routinely performs ESIs (Group A), vs those who had a clinical evaluation by a fellowship-trained spine specialist who referred the patient for the procedure to be done by a different physician (Group B).

**Study design/setting:**

Retrospective, observational, in vivo study of consecutive patients. Patient Sample. Patients undergoing cervical transforaminal (TF) or interlaminar (IL) ESIs at a single outpatient academic spine center.

**Outcome measures:**

Numeric Rating Scale (NRS) pain score improvement.

**Methods:**

Current procedural terminology (CPT) codes were used to search all consecutive patients who received a cervical TF or IL ESI between January 2010 and October 2018. All patients with pre and post-injection NRS pain scores within 60 days of the injection were included in the analysis.

**Results:**

A total of 363 ESIs were analyzed (178 ​TF and 185 IL). 275 patients were evaluated and referred for the injection by a spine specialist who performs these procedures (Group A), and 88 were evaluated and referred by a spine specialist who does not perform these procedures (Group B). Success was defined as > 50% improvement in the NRS pain score. 52% [95% CI: 47–57%] of all patients who received a cervical ESI achieved a successful outcome. There were better results in Group A with a 57% [95% CI: 51–63%] success rate compared to a 38% [95% CI: 28–48%] success rate in Group B. Group A also had a higher proportion of patients who achieved at least 80% pain relief (31% [95% CI: 26–36%]) compared to Group B (17% [95% CI: 9–25%]).

**Conclusion:**

This retrospective study demonstrated better results from cervical ESIs when patients were referred for the injection by a physician who performs ESIs. This research did not receive any specific grant from funding agencies in the public, commercial, or not-for-profit sectors.

## 1 Introduction

Patient selection and procedure planning are critical factors in the success of epidural steroid injections (ESIs) [1]. Prior to addressing the abundant procedural and safety nuances for the proper execution of an ESI, an important decision must first be made about whether the injection is indicated based upon the patient's clinical presentation and imaging findings [2]. Once this decision is reached, then, and only then, can the specific approach for the injection, based on the patient's site of pathology and particular anatomy, and the anticipated flow of injected medication from the selected needle location, be chosen.

Procedure planning is influenced by multiple variables including proceduralist training and experience, knowledge regarding comparative safety and effectiveness, and patient specific pathology and anatomic variables. Physicians who perform cervical ESIs consider many nuances when it comes to injection planning. First, the physician chooses between a transforaminal (TF) or interlaminar (IL) approach for the injection. This choice of technique is based upon the relative differences in effectiveness and safety. Regarding comparative effectiveness, the literature is sparse. The lumbar spine literature on this topic is more robust, with several studies showing better results from TFESIs [[3], [4], [5], [6]]. In the cervical spine, early data shows mixed results [7,8]. For TF injections, the location of the patient's pathology influences the level chosen for the injection, as foraminal pathology would typically be treated at the level of pathology, yet a more central process may be approached from the level below. For IL injections, the Multisociety Pain Workgroup (MPW) recommends that injections preferably be performed at the C7-T1 level, and not above C6-7 [9]. Additionally, the choice of technique is not limited to a decision between TF vs IL, but also extends to choices within the specific technique. For TF injections, a more obtuse angle of approach is associated with higher rates of ideal contrast flow patterns [10], and needle placement within the foramen also appears to influence contrast flow [[11], [12], [13], [14]].

Safety concerns from cervical ESIs have been studied and reviewed extensively over the previous few decades [15]. For TF injections, ideal needle placement is within 2 ​mm of the vertebral artery in 29% of patients, with foraminal stenosis and disc height loss being associated with vertebral artery proximity [16]. Due to the potentially devastating complications from inadvertent intravascular injection of most steroids during these procedures, the MPW recommends routine use of multiple safety practices including use of nonparticulate steroids during cervical TFESIs [9]. While several practitioners have suspected that particulate steroids result in better outcomes, studies have shown similar outcomes from dexamethasone compared to particulate steroids during TF injections [[17], [18], [19]]. Lastly, for IL cervical ESIs, the contralateral oblique fluoroscopic view has been recommended to further decrease the risk from these procedures [20].

ESIs are performed by physicians from multiple different specialties, with the majority being performed by anesthesiologists and physiatrists [21], and others done by radiologists, orthopedic surgeons, neurosurgeons, general surgeons, psychiatrists, and likely others [22,23]. It is common for ESIs to be ordered by physicians who do not routinely perform these procedures and for whom the nuances of ESI patient selection and target selection are less well known. It is currently unknown if better outcomes are achieved when patients are selected for the procedure by a physician who performs these injections. This study was conceived to examine differences in cervical ESI outcomes when comparing ESIs ordered by spine specialists who commonly perform these procedures to ESIs ordered by spine specialists who do not.

## 2 Material and methods

Institutional Review Board (IRB) approval was obtained from our academic medical center's IRB (IRB#48537) for a single site retrospective observational study and the study was conducted according to the Declaration of Helsinki. A waiver of consent was granted by the IRB. Current procedural terminology (CPT) codes 62321, 62310 (cervical/thoracic interlaminar epidural injection) and 64479 (cervical transforaminal epidural injection) were used to search the electronic medical record for eligible patients who received cervical epidural injections from any of the institution's four fellowship-trained interventional spine physiatrists – all practicing according to Spine Intervention Society Guidelines.

All consecutive patients, aged ≥18 years, between January 2010 and October 2018, were identified and included if receiving a first-time cervical ESI. Exclusion criteria included absence of an MRI in the electronic record obtained within 12 months prior to the injection, and lack of NRS pain score data available in the record both before and within 60 days after the ESI. Additionally, patients who underwent a diagnostic selective nerve root block without steroid were excluded.

During the timeframe of this study, patients in our center with cervical radicular pain were scheduled for their initial clinic evaluation with the next available provider regardless of subspecialty (spine physiatrist or spine surgeon), unless a preference was specified by the patient or referring provider. Both surgeons and physiatrists could refer patients for epidural injections. When referred by the physiatrist, the patient was most often scheduled with the referring provider but was allowed to schedule with any of the other spine physiatrists according to the patient's scheduling preference. When referred for an epidural injection from the surgical clinics, the patient was scheduled directly for the injection based on time preference without a clinic visit or screening by the physician performing the injection. In each instance, the ordering physician completed a form specifying the location and type of ESI recommended. This form was used for patient scheduling and surgery center preparation on a separate day. For each injection, the recommended injection was reviewed, however the final decision on the type, level, and side of the injection was determined by the physician performing the injection based on a pre-procedure evaluation of the patient and review of the MRI findings on the day of the injection. All injections were performed under fluoroscopic guidance following the Spine Intervention Society Practice Guidelines [1]. Dexamethasone was used for all TFESIs and methylprednisolone was used for all ILESIs.

### 2.1 Theory

We hypothesize that patients referred from spine specialists who routinely perform cervical epidural steroid injections will have better results than patients referred from spine specialists who do not perform these procedures.

### 2.2 Statistical analysis

Success in the primary outcome was defined as a Numeric Rating Scale (NRS) pain score improvement of ≥50% from pre-to post-injection. The proportion of patients who achieved this favorable response was calculated using baseline and follow-up encounter NRS pain scores, and 95% confidence intervals were calculated. Success rates were compared between patients who were evaluated and referred for the injection by a spine specialist who performs these procedures (Group A), and patients who were evaluated and referred by a spine specialist who does not performs these procedures (Group B). Similar calculations were performed for patients who achieved ≥80% pain relief.

NRS scores were non-normally distributed and therefore best presented as medians with interquartile ranges for descriptive statistics and box plots for visualization. Differences in pre- and post-procedure scores between Group A and Group B (overall and separately by TFESI and ILESI) were assessed with Mann Whitney U-tests. Differences in the change from pre-to post-procedure were assessed similarly. The pre-post change within each group was assessed with Wilcoxon signed ranks tests. To evaluate the combined effect of group and injection type on the magnitude of the pre-post change, a multivariable generalized estimating equation (GEE) including group, injection type, and their interaction (group∗injection type) were evaluated as variables. All analyses were performed in SAS v. 9.4 (Cary, NC, USA) with a two-sided level of significance of α ​= ​0.05.

## 3 Results

Before the epidural injection, patients were evaluated in clinic by either 1 of 4 fellowship-trained interventional spine physiatrists who routinely perform cervical ESIs (Group A), or by 1 of 9 surgeons who do not perform these procedures (Group B). 85 of the 88 injections in Group B were referred from fellowship-trained orthopedic spine surgeons, 2 were referred from orthopedic sports surgeons, and 1 was referred from a fellowship-trained spine neurosurgeon.

Overall, 571 patients met search criteria (316 who received a TFESI and 255 who received an IL ESI). A total of 208 patients (138 TFESI and 70 ILESI) were excluded due to lack of follow-up NRS data, follow-up data after 60 days, or no imaging within 1 year. 363 patients remained and were included in the analysis. 275 patients were in Group A, and 88 were in Group B. 178 injections were TF ESIs (114 in Group A, 64 in Group B) and 185 injections were IL ESIs (161 in Group A, 24 in Group B). Median baseline pain scores were similar in the two groups (6/10 in each group). The mean follow-up time for patients who received a cervical TFESI was 21 days, and 23 days for patients who received an ILESI. For the primary outcome of ≥50% reduction in NRS pain score, the overall success rate for all subjects was 52% [95% CI: 47–57%]. Group A achieved statistically significantly better results with a 57% [95% CI: 51–63%] success rate compared to a 38% [95% CI: 28–48%] success rate in Group B. Group A had trends towards better success rates than Group B for both TFESIs (50% [95% CI: 41–59%] vs 38% [95% CI: 26–50%]), and for ILESIs (62% [95% CI: 55–69%] vs 38% [95% CI: 14–62%]) (Table 1). Group A also had statistically better results in achieving ≥80% pain relief (31% [95% CI: 26–36%] vs 17% [95% CI: 9–25%] in Group B) (Table 2).

**Table 1:** Patients with a successful outcome, defined as ≥ 50% reduction in NRS pain score.

	Group A (referred by physician who performs ESIs)	Group B (referred by physician who does not perform ESIs)
Overall	57% [95% CI: 51–63%]	38% [95% CI: 28–48%]
Transforaminal ESI	50% [95% CI: 41–59%]	38% [95% CI: 26–50%]
Interlaminar ESI	62% [95% CI: 55–69%]	38% [95% CI: 14–62%]

ESI ​= ​epidural steroid injection; 95% CI ​= ​95% confidence interval.

**Table 2:** Patients with a successful outcome, defined as ​≥ ​80% reduction in NRS pain score.

	Group A (referred by physician who performs ESIs)	Group B (referred by physician who does not perform ESIs)
Overall	31% [95% CI: 26–36%]	17% [95% CI: 9–25%]
Transforaminal ESI	21% [95% CI: 14–28%]	11% [95% CI: 3–19%]
Interlaminar ESI	38% [95% CI: 31–45%]	33% [95% CI: 14–52%]

ESI ​= ​epidural steroid injection; 95% CI ​= ​95% confidence interval.

Group A showed a trend towards better results from ILESIs over TFESIs (62% [95% CI: 55–69%]) vs 50% [95% CI: 41–59%]), while there was no difference in success rates from ILESI vs TFESI in Group B (38% [95% CI: 14–62%] vs 38% [95% CI: 26–50%]). Patients in Group A who had an ILESI were more likely to achieve ≥80% pain relief (38% [95% CI: 31–45%]) compared to those in Group A who had a TFESI (21% [95% CI: 14–28%]). Group B showed a trend towards this (33% [95% CI: 14–52%] vs 11% [95% CI: 3–19%]) (Table 2). Outcomes of TF vs IL ESIs in this population is the topic of another article under preparation.

**Fig. 1:**
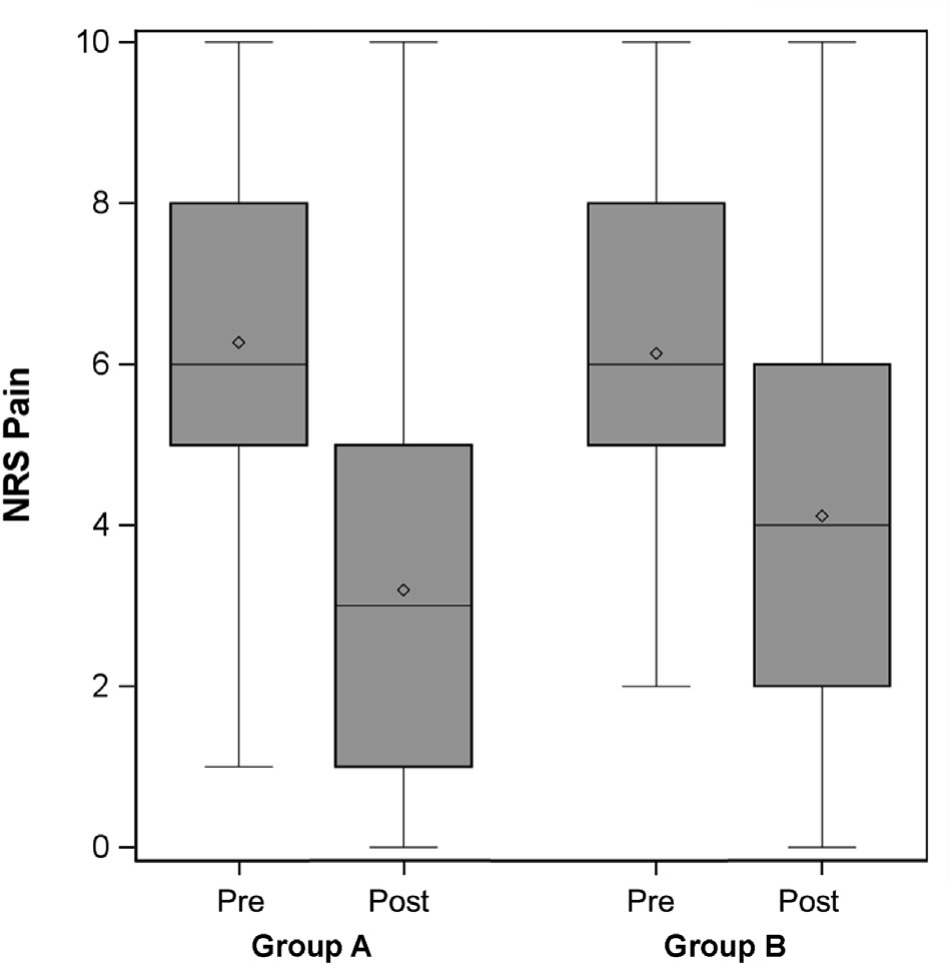
Median NRS pain scores pre and post injection, all injections.

**Fig. 2:**
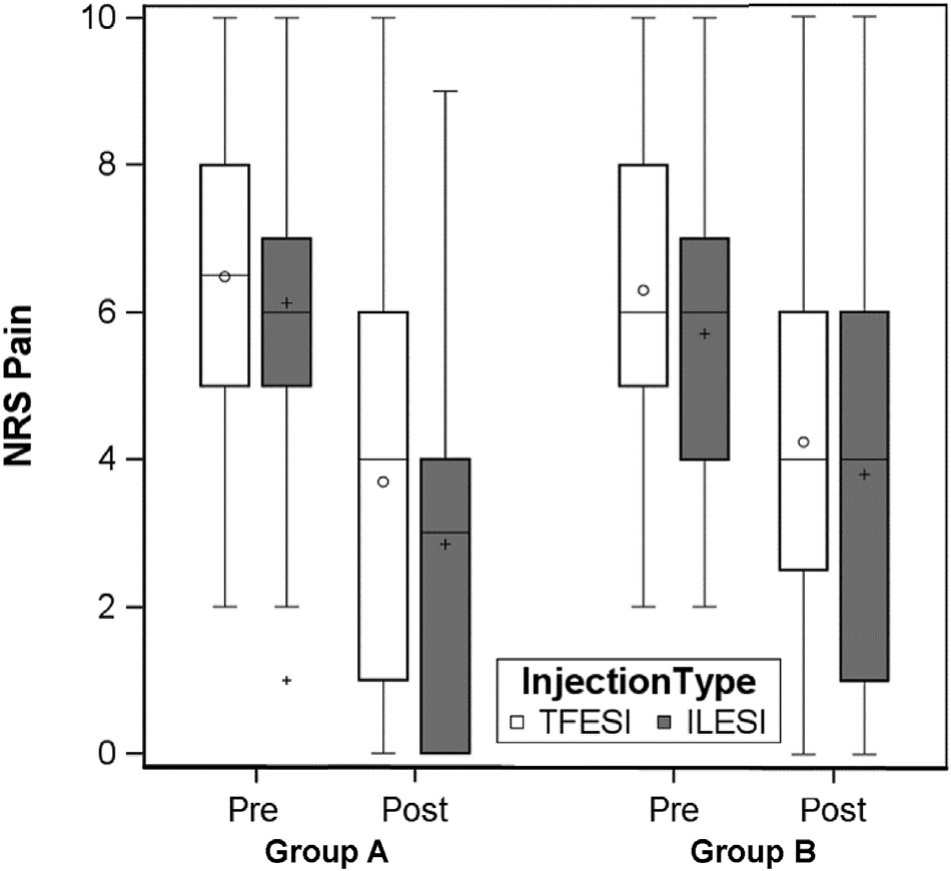
Median NRS pain scores pre- and post-injection by injection type.

Median NRS pain scores improved for both groups from pre-to post-injection (p ​< ​0.001 for both), but improvement in NRS pain score was greater in Group A compared to Group B (6–3 vs. 6 to 4, p ​< ​0.001) (Fig. 1). There was greater improvement in NRS pain score in group A compared to group B for patients who received an ILESI (6–3 vs. 6 to 4, p ​= ​0.01), and a trend towards greater improvement in NRS pain score in group A compared to group B for patients who received a cervical TFESI (6.5–4 vs. 6 to 4, p ​= ​0.055) (Fig. 2). In the multivariable model, the magnitude of the change in NRS scores from pre-to post-injection differed by group (A vs. B, p ​= ​0.003), but not by injection type (p ​= ​0.18). The difference between Groups A and B did not differ based on injection type (p ​= ​0.33).

## 4 Discussion

Patient selection is a key factor in determining success from medical treatments. The purpose of our study was to determine if physicians who are trained in and routinely perform a procedure have higher success rates than those who refer patients for the procedure, but do not perform it themselves. Since it is commonplace for patients to have ESIs recommended by a spine specialist who does not routinely perform these procedures, and then performed by a physician who does perform these procedures, this scenario provided an ideal case study for our question.

Our results demonstrate that patients referred from spine specialists who are trained in and routinely perform cervical ESIs have better outcomes compared to patients referred from spine specialists who do not perform these procedures. It is unclear if these outcome differences were due to patient selection or due to the choice of which procedure was performed. However, if the physician performing the procedure felt that a different technique (TFESI vs ILESI) or spinal level was the most appropriate procedure to perform on a patient who was referred from one of the physicians who does not perform these procedures, the performing physician typically changed the procedure to what they felt was most appropriate. Therefore, the outcome differences were most likely due to patient selection.

Our results are not unexpected. It is rational to suspect that physicians who are trained in a specific treatment would be best qualified to select patients for that treatment. The amount of literature on epidural steroid injections (ESIs), including studies on technique, effectiveness, and complications, has grown exponentially over the past two decades [24]. Physicians who perform these procedures are most likely able to stay up to date on the growing body of literature, and to understand the multiple nuances about effectiveness, safety, and optimal location for that procedure.

One strength of our study is that we had a large sample size of data collected prospectively on consecutive patients with similar baseline NRS scores. Four fellowship trained interventional spine physiatrists and 7 spine surgeons were involved in the evaluation of patients (an additional 2 non-spine orthopedic surgeons recruited 2 out of the 88 patients referred from the surgical clinics). This large number of physicians limits the biases that could be present from the practice patterns of a single physician.

After the timeframe of this study, we instituted a required physiatry video visit prior to a scheduled ESI when the injection is requested by a spine specialist who does not perform these procedures. Anecdotally, these video visits commonly result in changes to the injection plan, including changes to the ESI technique or spinal level, cancelling the ESI, or changing to a diagnostic intervention targeting axial neck pain. In a future study, we hope to compare outcomes prior to and after this policy change to determine its impact on outcomes in Group B. Additional future research should investigate the factors responsible for the observed differences to help develop policies that can further improve ESI outcomes.

Our study has limitations. The study was retrospective and open to many forms of bias. For example, we only included patients for whom pre- and post-injection NRS data was available. It is possible that patients in Group B who had successful outcomes were less likely to follow-up and were therefore excluded from this study. In addition, patients who were seen in surgical clinics may have more complex or severe pathology that was less likely to respond to an epidural steroid injection, however the similar baseline NRS pain scores suggests that this was not the case. Also, the surgical and physiatry clinics are in the same physical location, and no clinical screening criteria are used to guide patients towards one specialty over the other. It is also possible that patients enter into an ESI with different expectations when recommended by surgeons versus physiatrists, and this could have an impact on outcomes. It is well-known that the placebo response from injections is robust, and patients may have more confidence in the procedure when it is performed by the physician who recommended it to them in clinic. Since patients in Group A spent time in clinic and developed a more meaningful relationship with the physician who performed the injection, they may have had more trust in that physician and a stronger desire to validate the well-meaning efforts of that physician. This could have led to unconscious bias on the part of these patients to experience better outcomes. Another limitation is that our study only measured improvement in NRS pain score and did not evaluate other outcomes such as functional status or subsequent surgical treatment. Additionally, regional rates of surgical fusion have been shown to have a 20-fold variation, representing the largest coefficient of variation of any surgical procedure [25]. Given the wide variation in spine practice patterns, our findings may not be generalizable to other populations of patients and physicians. Another potential limitation to our study is that a larger proportion of patients in Group An underwent ILESIs compared to Group B. While Group A did show higher success rates in achieving the secondary outcome of ≥80% improvement in pain score from ILESIs compared to TFESIs, no statistically significant differences in outcomes were seen between the two techniques in Group B. Further analysis of our data comparing ILESI vs TFESI is currently underway. Lastly, different steroids were used for the two types of injections – dexamethasone for TFESIs and methylprednisolone for the ILESIs. Previous studies have shown no significant outcome differences when comparing dexamethasone to particulate steroids during both cervical [17] and lumbar TFESIs [18,19]. Also, patients in group B had identical success rates from the two types of injections. So, while we suspect that the different steroids did not significantly influence the results of our work, it cannot be entirely excluded, and this warrants further investigation. Additional studies can address these questions further.

## 5 Conclusion

Our retrospective analysis demonstrated better results from cervical epidural steroid injections in patients referred for the injection from a spine specialist who routinely performs these procedures, compared to patients referred for the injection from a spine specialist who does not.

## Declaration of competing interest

The authors declare that they have no known competing financial interests or personal relationships that could have appeared to influence the work reported in this paper.
